# Electroresponsive Strategies to Combat Bacterial Infections

**DOI:** 10.34133/research.1116

**Published:** 2026-01-23

**Authors:** Yanni Song, Bingxiang Li, Heng Dong, Shengke Li, Dongliang Yang

**Affiliations:** ^1^ Key Laboratory of Flexible Electronics (KLOFE) and Institute of Advanced Materials (IAM), School of Physical and Mathematical Sciences, Nanjing Tech University (NanjingTech), Nanjing 211816, Jiangsu, China.; ^2^ State Key Laboratory of Flexible Electronics (LoFE), College of Electronic and Optical Engineering & College of Flexible Electronics (Future Technology), Nanjing University of Posts & Telecommunications, Nanjing 210023, Jiangsu, China.; ^3^ Nanjing Stomatological Hospital, Affiliated Hospital of Medical School, Institute of Stomatology, Nanjing University, 30 Zhongyang Road, Nanjing 210008, Jiangsu, China.; ^4^ School of Life Sciences, Nantong University, Nantong 226019, Jiangsu, China.

## Abstract

Drug-resistant bacterial infections threaten global health and economies. Stimulus-responsive antibacterial materials are promising for combating resistance and improving treatment precision. Among them, electroresponsive strategies stand out for being practical, economical, tunable, and safe. This perspective examines various strategies, including electroluminodynamic therapy, electrodynamic therapy, and electrocontrolled drug release, which enable precise antibacterial action with minimal side effects and demonstrate strong application potential. Finally, we outline current challenges and future directions to guide further research and practical applications.

## Introduction

Bacterial infections pose a substantial burden on global healthcare systems and have led to substantial numbers of fatalities [1–3]. The spread of drug-resistant bacteria seriously jeopardizes global public health and considerably undermines the efficacy of existing antibiotics [4–6]. Although the use of high-dose antibiotics is effective for temporarily inhibiting bacteria, it poses a risk of toxicity to healthy tissues [7]. Thus, there is an urgent need to develop novel antibacterial agents that enable precise and targeted treatments.

**Fig. 1.**
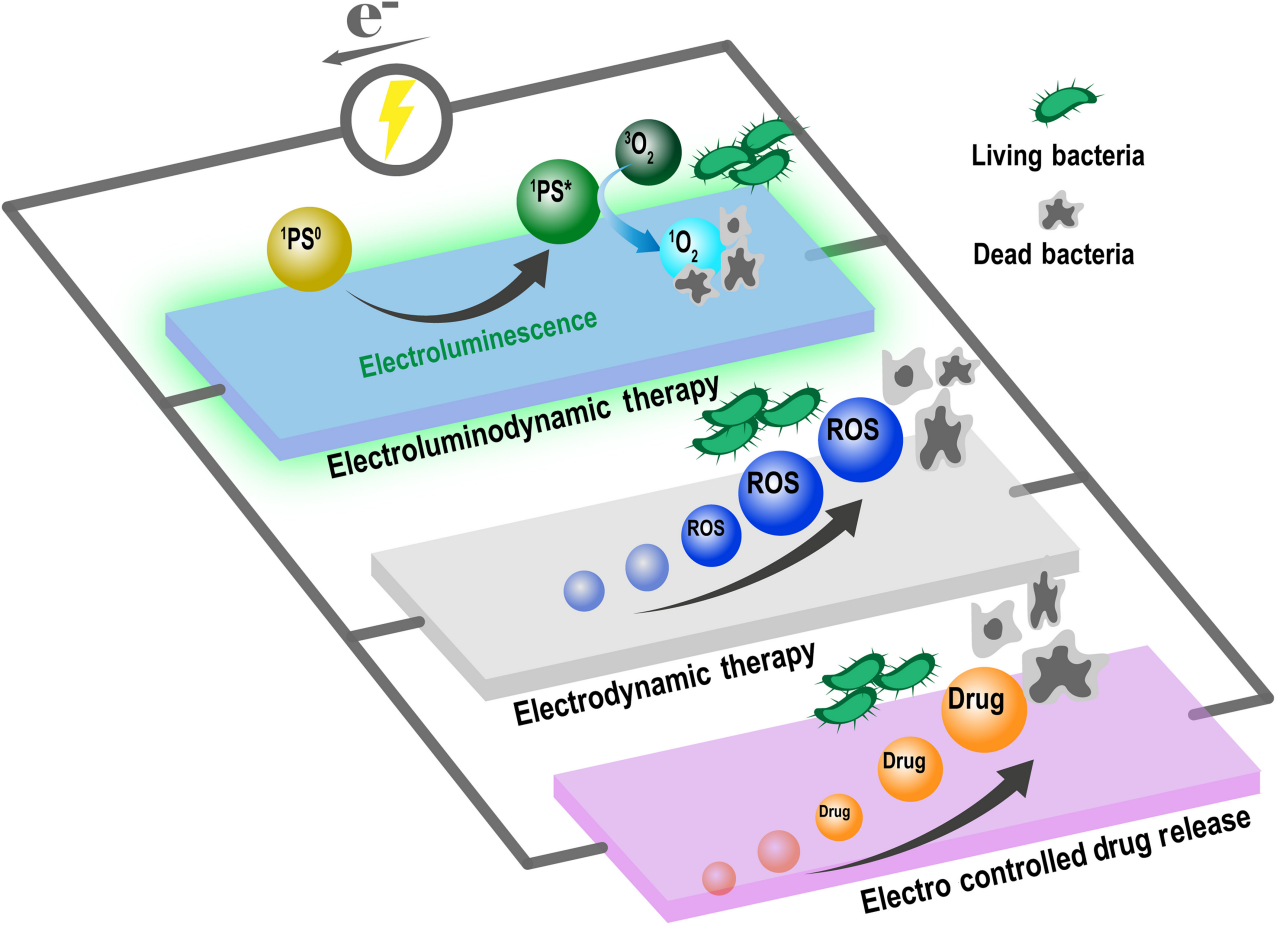
Electroresponsive strategies (i.e., ELDT, EDT, and electrocontrolled drug release) to combat bacterial infections.

Stimulus-responsive antibacterial materials can be activated by the endogenous or external triggers such as light, heat, or electricity to enable on-demand, spatially controlled therapy [8]. This approach enhances antibacterial efficacy while improving biosafety and minimizing off-target exposure, thereby slowing the development of resistance. Among these triggers, electrical stimulation is particularly advantageous because of its low cost, safety, ease of control, and ability to penetrate deep tissue—even reaching subcutaneous sites [9,10]. Therefore, various electrosensitive materials, including electroluminescent, conductive, and electrocatalytic substances, have been used to develop advanced antibacterial platforms [9]. In this perspective, we first outline the challenges in bacterial infection treatment and then summarize recent advances in three electrodriven strategies: electroluminescent dynamic therapy (ELDT), electrodynamic therapy (EDT), and electrocontrolled drug release systems (Fig .1), Last, the future prospects and ongoing challenges in this field are outlined.

## Electrodriven Antibacterial Strategies

### Electroluminescent dynamic therapy

ELDT is an innovative treatment strategy that uses electroluminescent materials as a light source to activate a photosensitizer (PS), generating reactive oxygen species (ROS) to achieve antibacterial effects [11]. In 2018, Wang’s team [12] pioneered the use of electrochemiluminescence (ECL) to replace external light for exciting PSs, enabling efficient bacterial inactivation. In this system, an electric field activates the ECL reagent (i.e., 5-amino-2,3-dihydro-1,4-phthalazinedione), whose emitted fluorescence excites the cationic PS oligomeric paraphenylene vinylene, producing ROS for sterilization. To enhance practicality, the system was integrated into a polyacrylamide (PAA) hydrogel, forming a flexible and durable antibacterial platform. However, the study was limited to in vitro assays and did not evaluate the in vivo effects. In 2022, Li’s team [13] further optimized and improved the system. They assembled rubrene (Rub) and rose bengal (RB) into nanoparticles via π–π stacking. When electrically excited, Rub emits light that activates RB, generating bactericidal ROS. The nanoparticles were embedded in a sodium alginate/PAA hydrogel integrated with a flexible battery, forming a conformable wound dressing. This dressing effectively combated drug-resistant bacteria both in vitro and in vivo, showing promise for infectious wound care.

### Electrodynamic therapy

EDT is an emerging antibacterial strategy where electrosensitizers generate ROS under an electric field. Unlike photoresponsive systems, the electric field directly activates electrosensitizers, overcoming light’s poor tissue penetration for noninvasive deep treatment. These systems also integrate with electronics for real-time spatiotemporal control, thereby enhancing precision and clinical adaptability.

For example, He and colleagues [14] synthesized a photo/electroresponsive polythiophene polymer functionalized with selenoviologen (Ppse). To shorten ROS delivery distance to bacteria, they created a wound dressing with an antisandwich structure: a PAA hydrogel containing Ppse is used as sandwich antibacterial platform. This places the electrocatalytic layer externally, improving tissue contact. Under light and electrical stimulation, it activates both photodynamic therapy and EDT, generating sufficient ROS to inhibit *Staphylococcus aureus* and speed wound healing.

Currently, most electrosensitizers are inorganic. Recently, Xu and colleagues [15] found that a zirconium-based metal-organic framework (MOF) with porphyrin linkers acts as an effective electrosensitizer. To stabilize it under physiological conditions, they coated the MOF with ginger-derived extracellular vesicles (EVs). This coating gave a negative surface charge, reducing protein adsorption, prolonging circulation, and promoting targeted accumulation at infection sites while neutralizing toxins. Upon reaching the infected tissue, electric stimulation sensitized MOF@EV to generate ROS, eliminating *S. aureus* and treating abscesses.

To improve EDT, strategies beyond increasing ROS yield—such as combining it with other methods—can create synergistic effects. For instance, Zhang et al. [16] developed a red blood cell membrane (RBCM)-coated AgPt bionic platform. By leveraging an electronic compensation effect, the AgPt minimizes premature silver ion release, reducing toxicity. The RBCM coating extends circulation, targets infections, and neutralizes toxins. After accumulating at the site, electrical stimulation induced ROS generation, disrupted the RBCM, and triggered silver ion release for synergistic antibacterial application.

### Electrocontrolled antibacterial release

Electric-driven antibacterial release is achieved through several key mechanisms. Electroresponsive materials can swell or contract volumetrically under an electric field to modulate release. Alternatively, electroactive “gating” units, such as ferrocene, undergo redox-induced conformational changes to open pores. Another approach relies on changes in surface charge density, where electrostatic repulsion enables the controlled release of antibacterial [17].

Guo’s team [18] developed a dual electro-/pH-responsive hydrogel based on chitosan-grafted polyaniline and oxidized dextran, in which positive voltage stimulation reduces the polyaniline to decrease the hydrogel’s positive charges, thereby releasing the negatively charged drug. For wound infection prevention, Yang et al. [19] developed an electric antibacterial suture by polymerizing polypyrrole (PPy) and tannic acid (TA) on a chitosan/gelatin-coated silk thread. A negative voltage reduces the positive charge of PPy and decreases its ability to retain TA, enabling substantial TA release; a positive voltage oxidizes PPy, increasing the positive charge to retain TA via electrostatic adsorption. This design prevents burst release and enables precise, bidirectional control of the drug.

## Conclusion and Perspective

In summary, electroresponsive materials are promising for treating bacterial infections. An external electric field enables these systems to offer personalized therapy. Compared to other stimulus-responsive strategies, they have distinct advantages: (a) spatiotemporal control over drug release/activation for precise targeting, reduced off-target effects, and mitigated drug resistance and (b) inherent versatility that facilitates synergistic combination therapies.

Despite encouraging results, challenges remain. The side effects and variable efficacy across patients are underexplored, while electrical parameters need optimization. Most current biocompatible platforms remain in basic research; advancing them requires more efficient carriers. For instance, ELDT has limited material choices, and the electrocatalytic efficiency of electrosensitizers needs to be improved [20]. Furthermore, effectiveness against diverse bacteria requires evaluation to determine whether combination therapies are needed. Future efforts will concentrate on developing effective, broad-spectrum, electroresponsive antibacterial strategies.
